# Gait Analysis under Spatial Navigation Task in Elderly People—A Pilot Study

**DOI:** 10.3390/s21010270

**Published:** 2021-01-03

**Authors:** Natalia Pawlaczyk, Magdalena Szmytke, Michał Meina, Monika Lewandowska, Justyna Stępniak, Bibianna Bałaj, Joanna Dreszer

**Affiliations:** 1 Faculty of Philosophy and Social Sciences, Institute of Psychology, Nicolaus Copernicus University in Toruń, Podmurna 74, 87-100 Torun, Poland; pawlaczyk87@umk.pl (N.P.); mlewando@umk.pl (M.L.); bibianna@umk.pl (B.B.); 2 Faculty of Psychology, University of Warsaw, Stawki 5/7, 00-183 Warsaw, Poland; m.szmytke2@uw.edu.pl; 3 Department of Applied Informatics, Faculty of Physics, Astronomy and Informatics, Nicolaus Copernicus University in Toruń, Grudziądzka 5, 87-100 Torun, Poland; mich@is.umk.pl; 4 Centre for Modern Interdisciplinary Technologies, Nicolaus Copernicus University in Toruń, Wileńska 4, 87-100 Torun, Poland; justyna.stepniak3@gmail.com

**Keywords:** spatial navigation, gait, dual-task, aging

## Abstract

A decline in the Spatial Navigation (SN) abilities has been observed in the course of healthy aging. Walking is an inseparable part of the navigation process; however, research tasks overlook this aspect in studies involving seniors. The present study was designed to overcome this limitation by recording gait parameters during natural environment navigation and to determine gait indicators that most accurately assign the participants to the proper age category. Thirteen elderly (mean age = 69.1 ± 5.4 year) and sixteen young women (mean age = 21.5 ± 2.2 year) equipped with gait sensors were asked to learn a path while walking in a real building (Learning Phase), reproduce the path (Memory Phase) and reach targets after a 30 min delay (Delayed Phase). The Receiver Operating Characteristics (ROC) analysis showed that our self-developed Gait Style Change indicator, that is, the difference in the probability of feet landing between particular SN task phases, classified the participants into either the elderly or the young group with the highest accuracy (0.91). The second most important indicator, the Task-Related (step counts in each SN task phase), achieved the accuracy discrimination of 0.83. The gait indicators, comprising single gait parameters measured while navigating, might be considered as accurately differentiating older from younger people.

## 1. Introduction

Spatial Navigation (SN) is defined as a complex ability to find the right way in the environment, plan how to reach a particular destination, and return to the starting point [1,2]. SN recruits a wide range of cognitive functions [2,3,4,5] and also involves much of the brain, mainly the hippocampus, parahippocampal gyrus, prefrontal, and parietal cortices (see Reference [6] for a review). SN skills deficits, coexisting with changes in the brain regions mentioned above, are observed in the course of Mild Cognitive Impairment, Alzheimer’s Disease, or healthy aging [7,8,9]. SN has been typically evaluated using the multi-staged computer and virtual reality tasks [10,11] or the real (natural) environment [12,13]. The virtual reality technology allows studying SN in well-controlled conditions [14], but it also deprives participants of the possibility to naturally explore the surrounding through walking which might reduce the ecological validity of these methods [15,16]. Compared to computer tests, the real environment tasks where a predefined path is required to be retraced seem to be closer to daily living situations and much more suitable for the seniors unfamiliar with information technology. Furthermore, when we take into account that SN is based on two kinds of information: visual and that delivered from the vestibular signals and motor efference copies [17], deprivation of the possibility to move in space while navigating, might result in committing more errors compared to the condition where visual cues are available and exploration by walking is possible [18].

Considering all the aforementioned reasons, in the present study, we decided to use the real environment task to investigate the SN ability of elderly people. When reviewing the contemporary research in this area, we have discovered that the existing measures do not capture all mental processes engaged in SN (e.g., uncertainty about decisions made on the path [12]). To fill this gap, we decided to record gait parameters during navigation. The rationale behind this idea comes from the evidence suggesting that some gait features are associated with cognitive processes [19,20,21,22,23,24], including those involved in SN, for example, executive functions (necessary to plan where to go and how to get there) [25,26,27] or attention and working memory [28,29]. Therefore, we claim that particular gait characteristics might be additional indicators of the real environment SN task performance.

Subtle changes in some gait characteristics are considered as predictors of cognitive decline [19,20,23,29,30,31], for example, an increase in stride-to-stride variability while usual walking and dual-tasking were found to be sensitive to dementia [29]. To date, several studies have revealed abnormalities in gait parameters in elderly people while walking at their comfortable speed, that is, a decrease in gait velocity and step length [32] or a reduction of cadence, step and stride length [33]. In healthy aging faster gait speed coexisted with better performance in memory, executive function, and global cognition tasks [20]. Furthermore, among older people both spatial (stride length) and temporal (e.g., gait speed, step count, cadence) gait features as well as greater variability in stride length, swing time, and stance time were related to deterioration in global cognition as well as in more specific domains such as memory, language, visuospatial and executive functions [23].

The relationship between cognitive demands and gait in healthy aging has been also tested with the use of a dual-tasking procedure where a cognitive task (e.g., talking, counting) is performed while walking (see Reference [28] for a review). In such a condition seniors (women mostly) demonstrated altered gait parameters compared to young adults, predominantly decreased gait speed and increased variability in stride velocity [34,35,36]. Furthermore, attention, and memory turned out to predict gait velocity in the dual-task condition in persons at the age of 70 and over [19].

Despite the fact that healthy older adults do not typically report serious spatial navigation problems, they are thought to perform more poorly, compared to young individuals, in the tasks requiring spatial memory in large-scale environments (see Reference [6] for a review). Specifically, age-related changes include impaired sequencing of route information [37], difficulty in the formation and use of the cognitive map to navigate [38] or inability to choose a proper strategy to deal with the SN tasks [39]. We believe that these subtle SN deficits might be well reflected in particular gait parameters registered during navigation. According to the authors’ best knowledge, this pilot study is the first demonstrating the effects of combining a real environment SN task and simultaneous gait monitoring.

In order to conduct the research in the ecological conditions, we developed an innovative procedure to investigate SN in the real building and equipped the participants with wearable sensors, previously developed by our team [40], to measure gait parameters while navigating. The goal of the present study was twofold: to develop a new procedure to examine spatial navigation ability in the real environment and to identify gait indicators, collected while navigating that would allow for the most accurate assignment of the participants to the proper category (young or elderly). We believed that this approach would allow us to determine whether our procedure could be sensitive enough to detect even subtle cognitive changes in the course of healthy aging.

Since elderly and young adults are proved to be different in terms of both SN tasks performance and gait characteristics [28,32,41,42,43,44], a classification analysis of particular gait features in the present study was conducted. Additionally, to reduce the variability of the analyzed parameters, only one sex (female) was included. Furthermore, we recruited only women characterized by a relatively high level of cognitive functioning to avoid a dramatic difference in SN task performance between young adults and seniors.

## 2. Materials and Methods

### 2.1. Participants

Out of 47 recruited women, 18 were excluded from analysis due to technical problems with the experimental procedure. The remaining 29 participants were assigned to two groups by age: elderly and youths (Table 1). Elderly participants were recruited from among students of the University of the Third Age in Toruń. The younger group consisted of students of the Nicolaus Copernicus University and randomly selected high schools in Toruń, Poland. All participants were in good general health, had no history of neurological/psychiatric diseases, or any other serious illnesses, and they did not use the medications affecting the Central Nervous System. They had normal hearing up to 8 kHz (verified by pure tone audiometry), normal or corrected-to-normal vision, and no self-reported limitations in walking. All women included in the study declared that they took regular physical exercise (three times a week).

The group of high-functioning elderly subjects was specifically selected to be best compared to their young counterparts. Their cognitive reserve was characterized by a high score of general cognitive functioning (MMSE ≥ 27) [45] no sign of severe depression (BDI ≤ 11) [46], educational attainment (≥11 years of education) [47], intellectual [48] and social activity in leisure time (or/and having tight social bonds) [49]. Moreover, age groups did not vary in years of education (U = 74, *p* = 0.46) and weight (U = 41, *p* = 0.1), however their height was significantly different (U = 23, *p* < 0.01), which is partially the effect of physiological aging on body characteristics [50]. The study was approved by the Ethical Committee at the Nicolaus Copernicus University Ludwik Rydygier Collegium Medicum and was in accordance with the Declaration of Helsinki. Each participant provided written informed consent to take part in the study after the procedure had been fully explained.

### 2.2. Study Design

The study protocol comprised two sessions, administered within a day and separated by a ca. 30-min break. First, each participant completed questionnaires concerning her health condition and habits, and then the MMSE and BDI were administered. During the second session, the Navigation Task (NT) was performed. Each session lasted ca. 1.5 h (with short breaks) and the duration of the session was adjusted to the participant’s individual work pace.

### 2.3. Navigation Task (NT)

The NT was conducted in the complex multi-level building of the Faculty of Physics, Astronomy and Informatics, Nicolaus Copernicus University in Toruń, Poland. Such a complex building was selected to reduce participants’ tendency to rely on cues available directly in the visual field (see Reference [51]). Each participant declared that they had never visited the place where the NT was conducted and were not familiar with the building’s route network.

The NT was adapted from Koening et al. [13] studies and consisted of three phases: (1) Learning Phase (LP), (2) Memory Phase (MP), and (3) Delayed Phase (DP). During the LP phase the participants were led by the experimenter along a predefined path containing 10 target locations. On the ground floor there were four objects (elevator, bar, whiteboard, photocopying shop), on the first floor there were two locations (columns, the poster on the wall), on the second floor there were two objects (telephone, computer room), and in the basement, there were two objects (tables). The experimenter provided each target’s name and the floor where it was located. Participants’ task was to remember each target and path leading to it.

Immediately after the LP, the MP was administered. Participants reproduced the previous presented path and pointed at the target locations along the way. The DP phase took place after a 30-min-break and required finding 10 targets, given one by one, and reaching them in the shortest possible way. These targets had been already used during the LP and MP and their order was counterbalanced across the participants. In order to guarantee their safety and to control the task performance, the experimenter followed each participant at the 2-m distance during both the MP and the DP phases. In case of going off the track participants were given a guiding cue [13]. During the task participants were asked to maintain their natural walking pace. The total path length was approximately 385 m for each phase.

### 2.4. Gait Measurement

While performing the NT each participant was equipped with two inertial measurements units (AltIMU-10 v4). The sensors were connected to custom-made integrated board that stored the data on SD card. Sensors were configured for acquisition of 6 degree-of-freedom data—three axis acceleration and angular rate (dynamic bandwith: ±8 g, ±2000°/s respectively) at 400 Hz sampling rate. Units were attached to the midpoint of the left and right metatarsi with elastic clamping bands. The inertial data were recorded separately for each foot and stored on an internal SD card. The process of data acquisition was managed and monitored online via the mobile phone application.

### 2.5. Gait Data Preprocessing

The motion sensors enabled the raw accelerometer data collection that was used to compute the traversing path of the subjects. An exact three-dimensional reconstruction of the feet movement was performed by the deduced navigation (DN) algorithm that integrated sensory readings (motion sensors attached to the feet). Technique zero-velocity update (ZUPT) was exploited to overcome sensor drift and measurement error [52]. The above operations allowed to determine a set of feet positions at the end of the stride phase, which is considered to be a series of heading normalized foot flat positions in relation to step starting position, further called Feet Landing (FL). This procedure enabled isolation of the individual gait parameters. Each of them was obtained separately for each leg (left, right) and phase of the NT (LP, MP, DP).

### 2.6. Gait Indicators

The single gait parameters (e.g., step count, length, time, and width as well as path length) were combined into several indicators. This approach was used because the classification analysis based on individual gait parameter did not bring a satisfactory solution (the mean significance of all single gait parameters was below 0.1). The gait measures were grouped into the following categories based on the literature: Task Related (parameters that correspond to the quantification of solving navigation task; step count and path length separately in each NT phase [16,53]); Gait Variability (standard deviations of spatial and temporal gait features, such as step length and step time [54] separately in each NT phase); and Mean Pace (mean values of step length and step time separately in each NT phase [28,55,56,57]).

**Figure 1:**
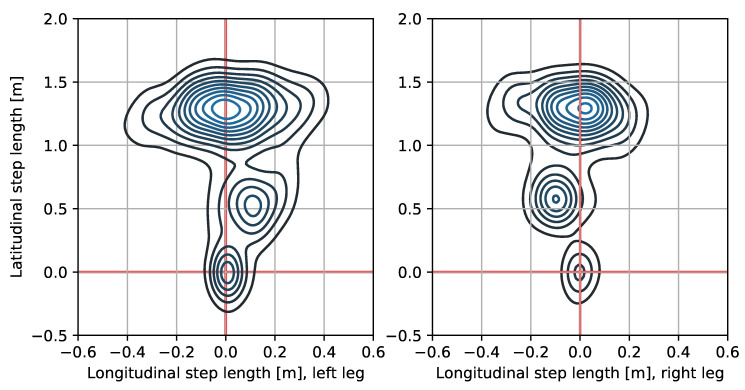
The example of Feet Landing Probability Distribution Function (Gait Style). This particular dataset includes three local maxima that depict three distinctive points of feet landing (based on the number of strides with particular length, and width). The shapes of the probability estimation consist of every subject but vary in the position of statistical moments and dispersions around those, resulting in different distribution in each task phase and across the subjects. We can see that the most frequent step length for the left foot was 1.5 [m] with a step width of 0.8 [m].

A novel indicator, developed specifically for our procedure, called Gait Style Change was used, as well. Gait Style, that is, a measure of the probability of foot landing when performing particular phases of the NT, is the number of steps of a given length and width. A graphic representation of Gait Style is shown in Figure 1. Consistently with the above definition, in the current work, the Gait Style Change is conceived as the difference in the probability of feet landing between particular phases of the NT. To estimate the probability of feet landing in each NT phase (Gait Style) we used multivariate Kernel Density Estimation with Scott’s rule for optimal bandwidth matrix selection [58]. That calculation allowed defining the Feet Landing Probability Distribution Function (FL-PDF). Then, the comparison of FL-PDF across the subjects and the NT phases was exploited by the Earth Mover Distance algorithm (EMD) [59]. That analysis defines a distance measure between two probability distributions for bivariate probabilities. It allows estimating the minimum cost of transforming one probability into another by comparing all of the moments of the density function and summarizing them as one distance value of Gait Style Change.

### 2.7. Data Analysis

Gait measurements of each category were combined into a single augmented indicator using the Principal Component Analysis (PCA). This approach allows for the correct comparison of the different gait parameters categories without being influenced by the dimensionality in each group (the number of definable parameters in each category is varied, see Table 2). During the generation of factor score for each created indicator, no rotation was used.

Binary classification task (target variable was the age group) was conducted using a support vector machine (SVM) classifier with linear kernel (C = 1.0). Each augmented indicator was tested separately using 5-fold cross-validation. To carry out the classification evaluation the Receiver Operating Characteristics (ROC) analysis was used. The ROC was performed to validate the procedure and find the best-fit age classification indicator, minimizing the number of misclassifications [60]. Moreover, the area under curve (AUC) of the ROC analysis was used to determine the probability of correct classification, where “1” was a theoretically perfect distribution to our age groups and “0.5” was a chance level. The accuracy of discrimination is considered to be on a low level when the value is between 0.5 and 0.7, the range between 0.7 and 0.9 is considered as a moderate level, and the value above 0.9 is a satisfactory point of high accuracy for group discrimination [61].

## 3. Results

There were 76,534 feet movements collected from both young adults (n = 16) and older people (n = 13), whereas 58,581 ( 76%) were classified as being part of the continuous walking routine (the main component of step landing probability distribution). Each participant performed 2570 ± 282 steps on average and the number of steps for each procedure was 856 ± 17. Therefore, the criterion of collecting >700 steps for proper gait measurement [56] was achieved. Since the information derived from the left-foot and right-foot sensors was not significantly different, only the parameters from the right foot were included in further analysis.

The PCA results with the factor loadings > 0.3, obtained separately for each indicator, are presented in Table 2. The Task-Related indicator received the highest inputs from the step counts in each phase of the SN task, the Gait Variability got the biggest loadings from both the standard deviations of step length and the step time in all the NT task phases. The Mean Pace was mostly loaded by the mean of step length in each task phase and the Gait Style Change indicator received the highest inputs from the FL-PDF differences between the LP and MP and between the MP and DP of the SN task. The ROC analysis (Table 3) revealed that only one indicator Gait Style Change) classified the participants into either elderly or young group with high accuracy (AUC = 0.91). The accuracy discrimination on the moderate level (AUC = 0.83) was achieved by the Task-Related indicator whereas the AUC values for the remaining groups of gait characteristics corresponded to a low discrimination ability.

## 4. Discussion

In the current work, we demonstrate a new procedure for investigating the SN in the real world. The novelty of our approach consists in combining a multi-stage task with gait features collected while navigating. Similarly to previous research [16,62], our SN task comprised subsequent phases differing in terms of cognitive load: the initial phase when participants learn the path by walking simultaneously with an experimenter is relatively easy whereas the other stages that require recalling the location of objects and retracing the path to them demand more cognitive effort. According to our knowledge, in none of such studies gait indicators have been determined.

The studies with the use of the dual-task paradigm indicate that the task difficulty might affect gait parameters [63]. Therefore, more complex tasks would cause a greater change in gait features than simple ones [19,64]. Congruently, we expected that the subsequent phases of our SN task and the amount of cognitive resources allocated to them might be reflected in particular gait parameters. The differences between both the SN performance and the gait characteristics could be especially observed among seniors demonstrating even subtle signs of cognitive decline [65].

In this study, the classification analysis was applied to assign elderly and young people to the proper age category based on gait indicators (see Table 3). This approach allows extracting the gait indicators sensitive to a subtle cognitive decline in healthy aging (our elderly group included women with high cognitive functioning only). The accuracy of each gait indicator in the classification of elderly and young people into the appropriate age category is discussed below.

### 4.1. Gait Variability and Mean Pace

The gait indicators that have been used in previous research were determined in our study and their accuracy in the classification of participants into particular age groups was evaluated. As a result, the Gait Variability turned out to have the lowest classification value. Despite that this indicator included the largest number of gait parameters, it classified participants into the proper age group by only random efficiency (51%). This effect is congruent with the literature and might result from large individual differences in the study samples (regardless of the chronological age) [29,41,66,67]. Specifically, participants are diverse in terms of a history of falls [66,67], the characteristics of the musculoskeletal system [41] and gait speed variability [29]. Furthermore, in these studies, various tasks have been applied, with different distances to be covered [44,68], and also various definitions of gait variability have been accepted [29,68]. The group recruited for the present study was not exceptional in these regards, being heterogeneous in terms of the aforementioned factors, which might explain a low classification value of our Gait Variability indicator.

The Mean Pace, based on the mean step length (Table 2), turned out to be a slightly better classifier with accuracy of 63% (Table 3). Previous evidence has demonstrated a decrease in step length with aging, especially in those persons who have experienced falls [69], which suggests that this gait parameter might reflect changes in the musculoskeletal system. Step length is also thought to be associated with gait speed [70,71,72], compensatory strategy in walking after falls [73], or problems in keeping balance [74]. Furthermore, the differences (or a lack of them) in gait speed (and possibly also in step length) between seniors and youth might be dependent on the experimental procedure, for example, walking in preferred or fast tempo, or without any control of it [75]. Therefore, following other authors [76] and based on our preliminary results we recommended taking into account various parameters that could potentially affect the step length while defining its relationship with the aging process.

### 4.2. Task-Related Indicator and Gait Style Change

In the present study, the Task-Related indicator showed a high, albeit not the highest, accuracy of the age-group classification (83%, Table 3). This gait indicator received the highest inputs in the PCA from the number of steps in each phase of the SN task (Table 2). Despite that path length was also investigated. Both aforementioned gait measures have been previously used to determine navigation accuracy [16,53]. Consistently with Tolman’s theory, a cognitive map is defined as a neural system for coding the representation of the explored environment in a third-person (allocentric) perspective [77]. Tolman’s observations led to the conclusion that the construction of a cognitive map allows for flexible behavior and use of alternative routes and shortcuts to the destination when the standard path is blocked. In our SN task, the number of steps may reflect the utility of this cognitive map in remembering the route. Participants while retracing the track and searching for the landmarks could get lost; hence, a greater number of steps occurred. Thus, the number of steps might be a valuable gait measure to differentiate between persons who deal with the spatial navigation tasks more or less successfully, for example, young adults and seniors, as it was demonstrated in our study.

Although the Task-Related indicator turned out to be quite a good classifier, we were not completely satisfied with the obtained solution. Fortunately, our procedure provided an opportunity to observe changes in gait parameters between particular stages of the SN task, which encouraged us to develop an additional gait indicator, that is, the Gait Style Change. It proved into classify our participants to particular age groups with the highest 91% accuracy (Table 3). Considering that gait is a complex and multidimensional process [25], we decided to build a measure that might reflect the whole stride. In our classification analysis, we used the Probability Distribution Function, which proved to provide the parameters of higher accuracy compared to the linear discriminant analysis [78]. We estimated the frequency of particular step types for each SN task phase based on their length and width (the variability of both the gait parameters proved to explain variance in gait performance in elderly people [55]). The Gait Style Change was obtained by subtracting the probability distribution of feet landing position between each phase of the NT. This subtraction allows investigating gait changes simultaneously in three dimensions, which practically means that one measure carries more information than an individually collected parameter. What is worth mentioning, this procedure through subtraction also eliminates individual differences in gait resulting from physical characteristics such as weight, height, or motor skills. For all the above reasons, the Gait Style Change might have turned out to be the most accurate in the classification of our participants into the proper age category.

## 5. Conclusions and Future Directions

The present work has showed the preliminary results of testing the accuracy of a multi-staged real-world spatial navigation task with simultaneous acquisition of gait parameters. The applied classification analysis revealed that two gait indicators, that is, the Task-Related one encompassing step count (in three separated phases of the navigation task) and the Gait Style Change built on subtraction of foot landing probability (again: in particular phases of the task) could classify young and senior women into the adequate age categories with the highest accuracy. Thus, we might carefully conclude that not single gait parameters but their compilations are better classifiers of the above groups. The obtained results (with 83% and 91% accuracy in classification to the group) are very promising enough that we may recommend considering them for investigating navigation abilities in natural environment tasks, especially to capture even subtle SN deficits like those observed in healthy aging.

Although in the existing studies in this area the authors have not taken into account the gait indicators composed of several characteristics, they have highlighted the importance of gait dynamics, that is, changes in its parameters over time. It has been shown that the gait analysis based on particular step characteristics and the dynamics of their changes could be a more precise indicator of task performance than the averaged results of gait parameters and also allows for accurate differentiation between children and adolescents, young and elderly, or those with and without a history of falls [79]. Therefore, we recommend building such indicators in further studies, each time when gait parameters are used to measure cognitive task performance. The proposed procedure of investigating navigation in the real environment might be also used in future studies to classify clinical groups, especially those presenting spatial perception and memory deficits (e.g., patients with Mild Cognitive Impairment or Alzheimer’s disease).

## Figures and Tables

**Table 1:** The characteristics of participants.

	Elderly Participants (n = 13)	Young Participants (n = 16)
Age years, mean (SD)	69.1 (5.4)	21.5 (2.2)
Education years, mean (SD)	12.8 (2.7)	13.6 (2.1)
Height centimeters, mean (SD)	160.4 (6.0)	167.0 (5.2)
Weight kilograms, mean (SD)	68.3 (7.3)	62.5 (10.3)

**Table 2:** The results of the Principal Component Analysis (PCA) analysis for each group of gait parameters (indicators).

Indicator	Gait Parameters	Coefficients
Gait Variability	SD of step length (MP)	−0.49
	SD of step length (LP)	−0.45
	SD of step time (DP)	−0.35
	SD of step time (MP)	−0.35
	SD of step time (LP)	−0.30
Mean Pace	Mean of step length (MP)	−0.61
	Mean of step length (LP)	−0.46
	Mean of step length (DP)	−0.45
Task-Related	step count (MP)	0.80
	step count (DP)	0.48
	step count (LP)	0.33
Gait Style Change	FL-PDF phase substraction (LP-MP)	0.87
	FL-PDF phase substraction (MP-DP)	0.40

Note: FL-PDF, Feet Landing Probability Distribution Function; LP, learning phase of Navigation Task; MP, memory phase of Navigation Task; DP, delayed phase of Navigation Task; NT, Navigation Task.

**Table 3:** Discriminative accuracy of each group of gait parameters (indicators).

Indicator	Area Under Curve	Confidence Interval	
		Lower	Upper
Gait Style Change	0.91	0.81	1.0
Task-Related	0.83	0.70	0.95
Mean Pace	0.63	0.47	0.81
Gait Variability	0.51	0.34	0.68

## Data Availability

The data presented in this study are available on request from the corresponding author.
